# Epigenetic Control of Viral Life-Cycle by a DNA-Methylation Dependent Transcription Factor

**DOI:** 10.1371/journal.pone.0025922

**Published:** 2011-10-11

**Authors:** Kirsty Flower, David Thomas, James Heather, Sharada Ramasubramanyan, Susan Jones, Alison J. Sinclair

**Affiliations:** 1 School of Life Sciences, University of Sussex, Brighton, United Kingdom; 2 Epigenetics Unit, Department of Surgery and Cancer, Imperial College, London, United Kingdom; 3 Infection and Immunity Division, University College London, London, United Kingdom; 4 The James Hutton Institute, Dundee, United Kingdom; George Mason University, United States of America

## Abstract

Epstein-Barr virus (EBV) encoded transcription factor Zta (BZLF1, ZEBRA, EB1) is the prototype of a class of transcription factor (including C/EBPalpha) that interact with CpG-containing DNA response elements in a methylation-dependent manner. The EBV genome undergoes a biphasic methylation cycle; it is extensively methylated during viral latency but is reset to an unmethylated state following viral lytic replication. Zta is expressed transiently following infection and again during the switch between latency and lytic replication. The requirement for CpG-methylation at critical Zta response elements (ZREs) has been proposed to regulate EBV replication, specifically it could aid the activation of viral lytic gene expression from silenced promoters on the methylated genome during latency in addition to preventing full lytic reactivation from the non-methylated EBV genome immediately following infection. We developed a computational approach to predict the location of ZREs which we experimentally assessed using *in vitro* and *in vivo* DNA association assays. A remarkably different binding motif is apparent for the CpG and non-CpG ZREs. Computational prediction of the location of these binding motifs in EBV revealed that the majority of lytic cycle genes have at least one and many have multiple copies of methylation-dependent CpG ZREs within their promoters. This suggests that the abundance of Zta protein coupled with the methylation status of the EBV genome act together to co-ordinate the expression of lytic cycle genes at the majority of EBV promoters.

## Introduction

Infection of human B-lymphocytes by Epstein-Barr virus results in the establishment of a latent state in which a highly restricted set of viral genes are expressed [1]. This is accompanied by extensive methylation of CpG motifs in non-expressed viral genes [2], [3], [4]. In response to physiological stimuli, such as engagement of the B-cell receptor, epigenetic silencing of the viral genome is overturned, resulting in widespread activation of viral gene expression and lytic replication [4], [5]. The expression of a subset of host genes is also altered during this period [6], [7], [8], [9], [10], [11], [12], [13], [14].

The switch between latency and the lytic cycle is orchestrated by the viral gene *BZLF1*, which encodes the protein Zta (also known as ZEBRA, BZLF1, EB1, or Z) [15], [16], [17]. Zta resembles the AP1 family of bZIP transcription factors but has a unique dimerisation domain and does not form heterodimers with cellular bZIP proteins [18]. Three classes of Zta DNA binding sites (Zta response elements (ZREs)) have been defined for Zta [19]. Class I ZREs include classical AP1-like recognition elements. However, some Zta binding sites contain a CpG motif and Zta has the unusual property of binding preferentially to these ZREs when they are methylated [20], [21], defining class II ZREs [19]. Remarkably, some CpG-containing ZREs are only recognized in their methylated form (class III ZREs) [19], [20], [21], [22], [23], [24], [25]. Methylation of the viral genome occurs during latency and has recently been shown to be required for EBV replication [3]. The ability of Zta to bind to methylated ZREs suggests that Zta may have a direct role in overriding the epigenetic silencing of the viral genome to activate expression of viral genes required for lytic replication.

The requirement for methylation at critical ZREs may also contribute to the establishment of latency during the immortalization of infected cells. The EBV genome is not methylated when it enters cells but the genome gradually becomes methylated during immortalization and the establishment of viral latency [3], [4]. Zta is transiently expressed during the early period immediately after infection and is required for efficient immortalization [3]. It is therefore essential that Zta should not activate the full lytic replication cycle at this stage. A plausible hypothesis to explain this is that expression of key lytic cycle genes are controlled by class III ZREs that do not function in their unmethylated form.

We developed a computational approach to identify candidate ZREs and applied it to a genome-wide analysis of the EBV genome that revealed many novel target loci. The implications of these data for the ability of EBV to evade epigenetic silencing of the host viral genome is discussed.

## Results

### Prediction of ZREs core sequences bound by Zta using PROMO

**Figure 1:**
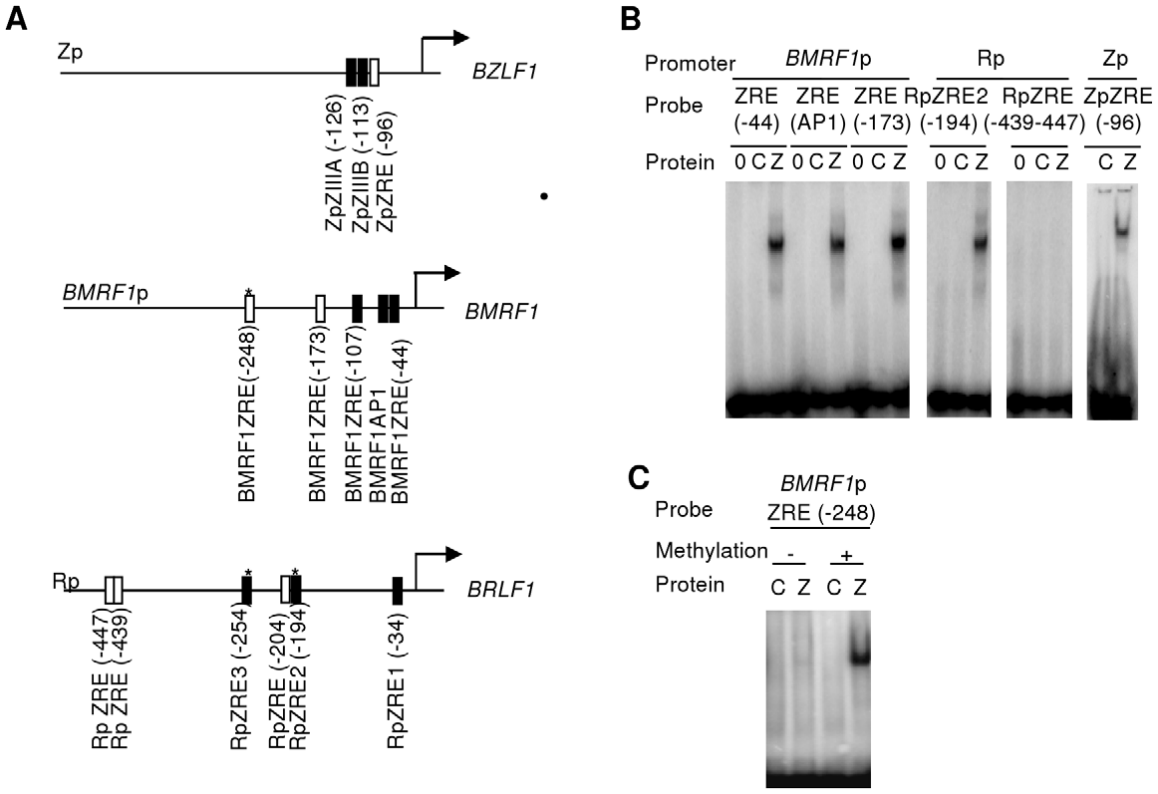
Evaluation of predicted ZREs in three EBV promoters. A. Summary of the known (filled box) and predicted (open box) core ZRE sequences within the proximal 500 nucleotides of the indicated *BZLF1*, *BRLF1 and BMRF1* promoters. The arrows represent the transcription start sites. Stars represent CpG ZREs. B. Double strand oligonucleotides were generated with the core ZRE sequence and at least 10 nucleotides of cognate sequence on either side. Following radio labeling, these were incubated with *in vitro* translated Zta and subject to EMSA. The reactions contained no protein, 0, control lysate, C or Zta, Z. The DNA probes are indicated above with their originating promoters. C. Double strand oligonucleotides were generated with the core ZRE sequence and 10 nucleotides of cognate sequence on either side. Following radio labeling, these were subject to *in vitro* methylation with SssI methyl transferase (+), or a mock reaction (−). Subsequently, they were incubated with *in vitro* translated Zta and subject to EMSA. The reactions contained control lysate, C; or Zta, Z.

In order to predict novel ZRE core sequences, we started by searching three well-characterized Zta-responsive promoters from the EBV genome (*BZLF1* promoter (Zp) [26], [27], [28], [29], *BRLF1* promoter (Rp) [27], [29], [30] and *BMRF1* promoter [31] using the PROMO algorithm [33], [34] and the position frequency matrix (PFM) for Tranfac 8.3 Zta transcription factor entry T00923 [32], [33]. These 3 promoters are known to contain eight previously verified sites: in Zp (ZREIIIA and ZREIIIB); in Rp (ZRE1, ZRE2 and ZRE3) and in BMRF1 promoter (AP1, ZRE(−44) and ZRE (−107)) (Figure 1 and Table S1) however, the PROMO algorithm only predicted one of these sites (RpZRE1) using the PFM T00923. In addition, 6 novel sites where predicted (Table S2).The ability of Zta to interact with each predicted site was assessed using electrophoretic mobility shift assays (EMSA) (Figure 1, Table S2), although three novel sites were identified, eight known sites were missed and three false positives were predicted indicating that the PFM used had a low sensitivity.

### Application of a novel ZRE PFM to predict CpG containing ZREs

**Figure 2:**
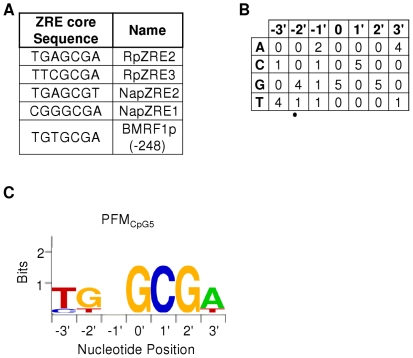
Position Frequency Matrix (PFM) of CpG containing ZREs (PFM_CpG5_). A. The core seven nucleotide element of the CpG containing ZREs found within the *BRLF1*, *BRRF1*, *and BMRF1* (Rp, Nap and BMRF1p) promoters are shown. B. The number of incidences of each nucleotide at each position and the thus the frequency were calculated. C. The consensus sequence is represented as a sequence LOGO.

**Figure 3:**
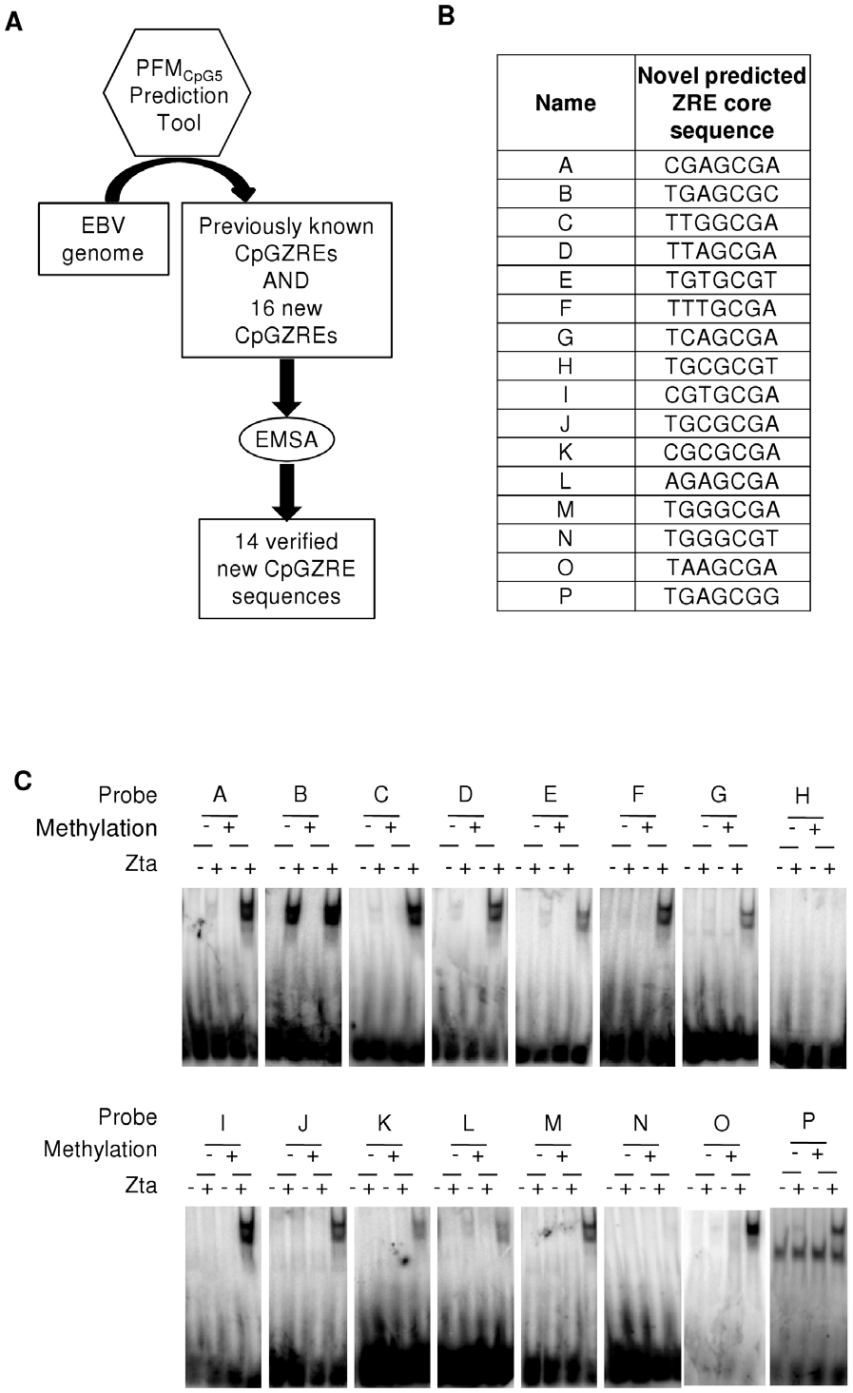
Zta recognition and methylation dependence of PFM_CpG5_ predicted CpG containing ZREs. A. Flow diagram illustrating the information flow from the PFM to the predictions of novel ZREs in the EBV genome and their subsequent evaluation. B. Core heptamer sequences, in both forward and reverse complement, of PFM_CpG5_ predicted CpG containing ZREs within the EBV genome. C. PFM_CpG5_ was used to predict the potential for further ZREs in the EBV genome. Double strand oligonucleotides were generated. Following radio labeling, these were subject to *in vitro* methylation with SssI methyl transferase (+), or a mock reaction (−). Subsequently, they were incubated with *in vitro* translated Zta and subject to EMSA. The reactions contained control lysate, C; or Zta, Z.

A new PFM was generated using the core sequences of five CpG-containing ZREs (denoted PFM_CpG5_) from the promoters described above and the *BRRF1* promoter [22] (Figure 2). The accuracy of the PFM was evaluated by searching for ZREs in the well-characterized viral promoters (Rp, Zp and *BMRF1*p). PFM_CpG5_ identified all 5 verified CpG containing sites and predicted two novel sites; one located in Rp, centered on −114, and one located in the *BMRF1* promoter, centered on −148. DNA binding experiments demonstrate that Zta interacts with both sites in a methylation-dependent manner, characteristic of class III ZREs (Figure 3), thus the new PFM (ZRECpG_5_) has a high level of sensitivity. The PFM_CpG5_ was then used to predict core ZREs in the complete EBV genome. Within the EBV genome a total of 16 novel sequence variants of CpG ZREs were predicted (A–P) (Figure 3). EMSAs were undertaken with each of the novel ZRE core sequences (both non-methylated and methylated) to evaluate Zta binding (Figure 3). All but two of the predicted ZRE sequences bound in the methylated form. Only one sequence bound significantly in the unmethylated form. Therefore 13 out of 16 predictions are classified as Class III ZREs, 1 is classified as Class II and 2 did not interact with Zta significantly. Combined with previously published ZREs, this resulted in a total set of 32 distinct sequence variants of ZREs (ZRE_32_) (Table 1).

**Table 1:** List of core ZREs included within ZRE_32_.

ZRE Core Sequence		Class	Names
Forward	Reverse		
TGAGCCA	TGGCTCA	I	Zp ZREIIIA
TGAGCCA	TGGCTCA	I	Rp ZRE1
TGAGCCA	TGGCTCA	I	Zp (−365)
TTAGCAA	TTGCTAA	I	Zp ZREIIIB
TGTGTAA	TTACACA	I	DSL ZRE1
TGAGCAA	TTGCTCA	I	DSL ZRE2
TGAGCAA	TTGCTCA	I	DSL ZRE7
TGAGCAA	TTGCTCA	I	BMRF1ZRE(−44)
TGAGCAA	TTGCTCA	I	BMRF1ZRE(−107)
TGTGTCA	TGACACA	I	DSL ZRE3
TGTGTCA	TGACACA	I	DSL ZRE4
TGTGTCA	TGACACA	I	DSL ZRE6
TGTGTCA	TGACACA	I	*DHRS9 ZRE1*
TGTGCAA	TTGCACA	I	DSL ZRE5
TGTGCAA	TTGCACA	I	*CIITA (221)*
TGAGTCA	TGACTCA	I	BSLF2+BMLF1
TGAGTCA	TGACTCA	I	BMRF1 AP-1
TGAGTCA	TGACTCA	I	*IL8 AP1*
TGAGTCA	TGACTCA	I	*DHRS9 (ZRE2)*
TGACTAA	TTAGTCA	I	Fp AP-1-Like Site
TGTGTCT	AGACACA	I	Zp (−96)
TGGCACA	TGTGCCA	I	BMRF1 (−173)
TGAGTAA	TTACTCA	I	*CIITA*
TGAGTAA	TTACTCA	I	*IL13*
GTTGCAA	TTGCAAC	I	*IL-8 ZRE*
GGAG**CG**A	T**CG**CTCC	III	Egr1
TGAG**CG**A	T**CG**CTCA	II	Rp ZRE2
TT**CGCG**A	T**CGCG**AA	III	Rp ZRE3
TGTG**CG**A	T**CG**CACA	III	BMRF1 (−248)
TGAG**CG**T	A**CG**CTCA	III	Nap ZRE2
CGGG**CG**A	T**CG**CCCG	III	Nap ZRE1
CGAGCGA	TCGCTCG	III	CpG5 A
TGAGCGC	GCGCTCA	II	CpG5 B
TTGGCGA	TCGCCAA	III	CpG5 C
TTAGCGA	TCGCTAA	III	CpG5 D
TGTGCGT	ACGCACA	III	CpG5 E
TTTGCGA	TCGCAAA	III	CpG5 F
TCAGCGA	TCGCTGA	III	CpG5 G
CGTGCGA	TCGCACG	III	CpG5 I
TGCGCGA	TCGCGCA	III	CpG5 J
CGCGCGA	TCGCGCG	III	CpG5 K
AGAGCGA	TCGCTCT	III	CpG5 L
TGGGCGA	TCGCCCA	III	CpG5 M
TAAG**CG**A	T**CG**CTTA	III	CpG5 O
TGAG**CG**G	C**CG**CTCA	III	CpG5 P

### Identification of ZRE core binding sequences in the EBV genome

**Figure 4:**
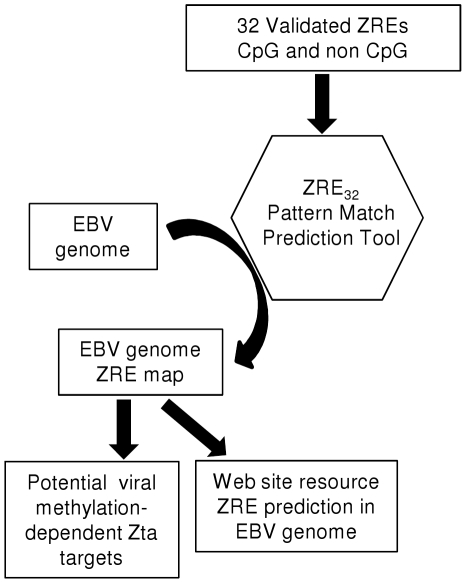
Information flow for the identification of ZREs in the EBV genome. Flow diagram illustrating the use of the pattern matching tool (ZRE_32_) to identify exact sequence matches for the 32 verified ZREs in the EBV genome.

Global analysis of the EBV genome was then undertaken using an exact pattern match with the 32 validated variants of the ZRE core sequence (Figure 4). This revealed 469 locations within the EBV genome that matched one of the ZRE core sequences (Table S3 and http://bioinf.biochem.sussex.ac.uk/EBV).

The occurrence of ZREs throughout the EBV genome appears to be widespread; with 81 out of 86 (94%) EBV promoters containing at least 1 ZRE core sequence (Table S4). This suggests that Zta has the potential to regulate the expression of the majority of EBV genes. Furthermore, 58 EBV promoters contained at least 1 CpG containing ZRE. These regions are methylated during latency [3], [4], suggesting that methylation-dependent Zta interaction with ZREs could influence the expression of a broad range of EBV genes once Zta is synthesized at the onset of lytic cycle.

Of particular relevance to the control of EBV gene expression immediately after infection are 22 EBV genes that contained CpG ZREs but have no methylation independent ZREs in their promoters (Table 2). These genes are prime contenders to be regulated in a strictly methylation-dependent manner by Zta. These were originally classified as displaying early lytic, late lytic and latent patterns of gene expression [34], but importantly, genome wide expression studies revealed that all are up regulated during lytic cycle in BL cells, with the majority reaching peak levels approximately 24 hours after lytic activation [6].

**Table 2:** EBV genes that contain CpG ZREs but have no methylation independent ZREs in their regulatory regions, with the kinetics and extent of any change in their expression in Akata cells undergoing lytic cycle [6].

Cycle	Promoter/Gene	Class	Sequence	Offset FromGene Start	Peakexpressiontime (hr)	fold changeexpression
early lytic	BALF1	II	TCGCTCA	−74	12	8.2
		II	TCGCTCA	−138		
		III	TCGCCCA	−383		
		III	ACGCTCA	−416		
late lytic	BALF4	III	TCGCGCA	−164	24	15.2
		III	TCGCTCG	−254		
		III	ACGCACA	−414		
unknown	BARF0	III	CGAGCGA	45	36	7.3
		III	TGCGCGA	−45		
early lytic	BARF1.2	II	TGAGCGA	114	24	9.2
		II	TGAGCGA	50		
		III	TGGGCGA	−488		
early lytic	BBLF2/BBLF3	III	TCGCCCA	91	12	19.5
early lytic	BBLF4	III	TCGCTCG	−221	12	7.9
		III	TCGCACA	−235		
		III	ACGCTCA	−357		
late lytic	BBRF1	III	AGAGCGA	−31	24	7
		III	TTCGCGA	−414		
late lytic	BBRF3	III	TGGGCGA	−34	24	20.5
		III	TTTGCGA	−177		
late lytic	BDLF1	III	TCGCCCG	−390	24	21.5
late lytic	BGLF2	II	TCGCTCA	133	24	14.3
early lytic	BGLF4	II	GCGCTCA	−260	8	12.1
		III	TCGCTTA	−316		
late lytic	BGRF1/BDRF1	III	CGGGCGA	−49	24	8.3
late lytic	BKRF2	II	TGAGCGC	155	24	14.1
		III	TTAGCGA	185		
early lytic	BKRF4	III	TTAGCGA	−70	24	16.3
		III	TGAGCGG	−246		
		III	CGTGCGA	−302		
early lytic	BLLF2	III	CCGCTCA	−18	24	12.4
	BOLF1	III	TCGCCCG	175	24	4.2
		III	TCGCTGA	−65		
late lytic	BORF1	III	CGGGCGA	130	24	12.4
		III	TGCGCGA	10		
		III	TGAGCGG	−20		
early lytic	BRRF1	III	CGGGCGA	−25	24	7
		III	TGAGCGT	−53		
late lytic	BTRF1	III	TGGGCGA	−292	24	8.5
unknown	BWRF1 repeats	III	CGGGCGA	72	∼	∼
		III	CGGGCGA	46		
		III	GGAGCGA	−422		
latent	Cp EBNAs	III	TGGGCGA	−70	24–48	6–8.2
		III	TTTGCGA	−204		
		III	GGAGCGA	−248		
latent	Wp EBNAs	III	TCAGCGA	180	48	6.1

**Figure 5:**
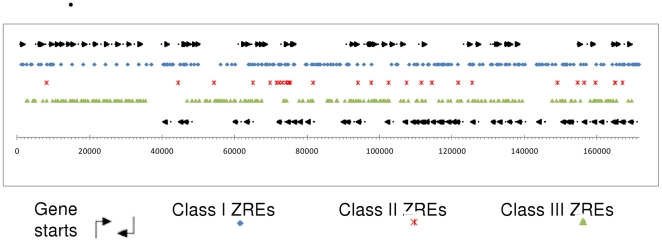
ZREs in the EBV genome. The entire EBV genome was subjected to an exact pattern match search, using the ZRE_32_ set of core sequences. Each site was classed by binding behaviour, and plotted by the first nucleotide of the site to form a genome wide map of ZREs. Class I sites are indicated by blue diamonds, Class II sites are indicated by red stars, Class III sites are indicated by green triangles, and gene starts and direction are indicated by arrows. The location of transcription start sites and their orientation are indicated by arrows.

**Figure 6:**
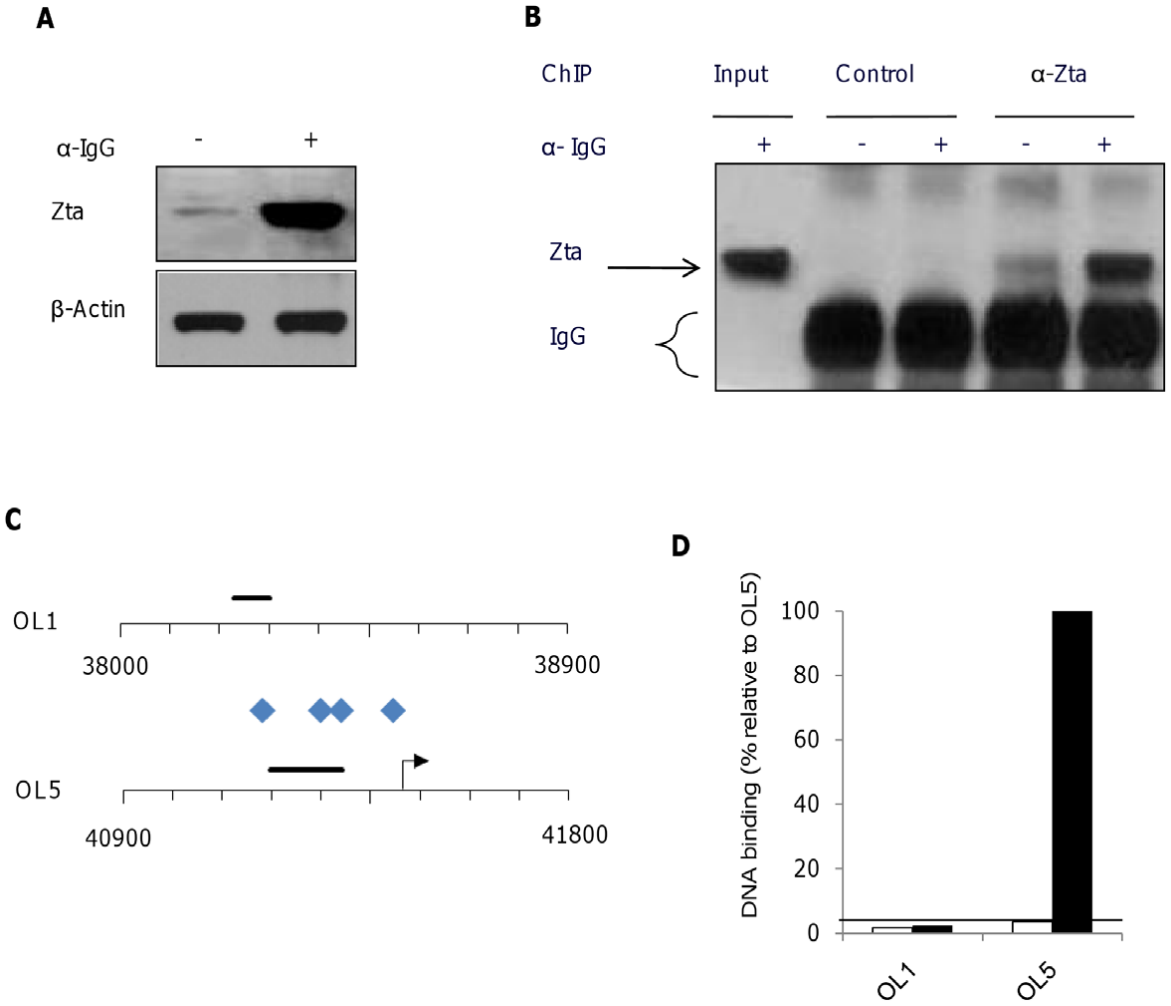
Chromatin precipitation (ChIP) analysis of Zta. Akata cells were induced or not to enter lytic cycle by the addition if anti-IgG as indicated. 48 hours later the cross-linking agent was added and chromatin and total proteins were harvested. A. The proteins were subject to western blot analysis for Zta expression. B. A chromatin precipitation experiment was undertaken with the Zta antibody and a control antibody. The precipitated chromatin and input chromatin were subject to western blot analysis to detect Zta protein. C. The location of ZREs and Q-PCR amplicons are illustrated for a region central to OriLyt left and for a region flanking OriLyt left. The genome co-ordinates are indicated. Transcription start sites and the direction of transcription are shown by arrows. Blue diamonds represents the class I ZREs. The amplicons used for Q-PCR are indicated as black horizontal bars. D. Association of Zta with OriLyt left was assessed using chromatin precipitation from Akata cells in lytic cycle, followed by Q-PCR. The binding is shown relative to maximal binding to Orilyt left.

**Figure 7:**
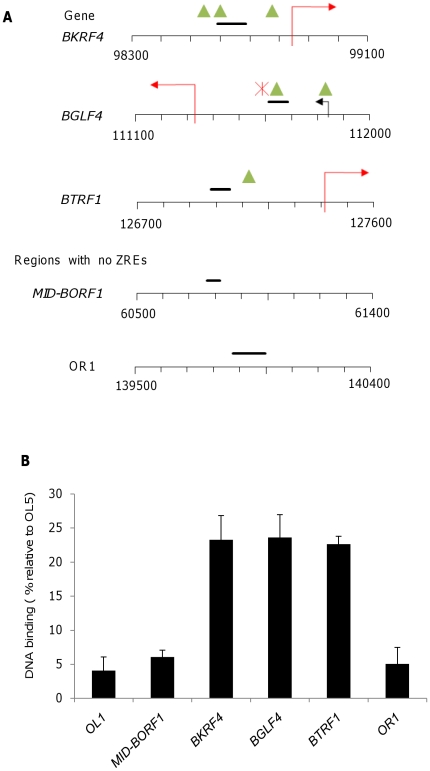
Chromatin precipitation (ChIP) analysis of Zta binding with the EBV genome. A. Five regions of the EBV genome are illustrated, together with gene names and sequence co-ordinates. Three contain core CpG ZREs and two do not. Transcription start sites and the direction of transcription are indicated with arrows. Green triangles represent class III CpG ZREs and the red star represents a class II ZRE. The amplicons used for Q-PCR are indicated as black horizontal bars. F. Association of Zta with the indicated regions of the EBV genome was assessed using chromatin precipitation from Akata cells in early lytic cycle (stalled prior to DNA replication with acyclovir), followed by Q-PCR. The binding is shown relative to maximal binding to OriLyt left (OL5).

Three of these promoters were chosen to question whether Zta interacts with the novel CpG ZREs *in vivo*; *BKRF4*, *BGLF4* and *BTRF1*. The location of the CpG ZREs in each promoter is indicated in Figure 5. Lytic cycle was activated in Akata cells [35] by surface immunoglobulin ligation, undertaken in the presence of acyclovir to inhibit genome replication. Chromatin was subjected to Zta immunoprecipitation (ChIP) and the interaction of Zta with these promoters was assessed by Q-PCR. The ability of this antibody to precipitate Zta bound to chromatin is demonstrated by western blotting in Figure 6. In addition, we show that Zta as opposed to a control antibody specifically precipitates chromatin from a region of oriLyt containing multiple ZREs. Using primer sets proximal to the CpG ZREs from *BKRF4*, *BGLF4* and *BTRF1* compared to primer sets from three regions of the EBV genome devoid of ZREs, we reveal that Zta specifically binds to all three of these promoters that contain novel CpG ZREs *in vivo* (Figure 7).

## Discussion

Following several iterations of a predictive and evaluative approach, we identified a set of 32 distinct sequence variants in the core 7-nucleotide sequence to which Zta can bind. This includes 20 variants containing a CpG motif, the majority of which (90%) are only recognized by Zta when they are methylated.

**Figure 8:**
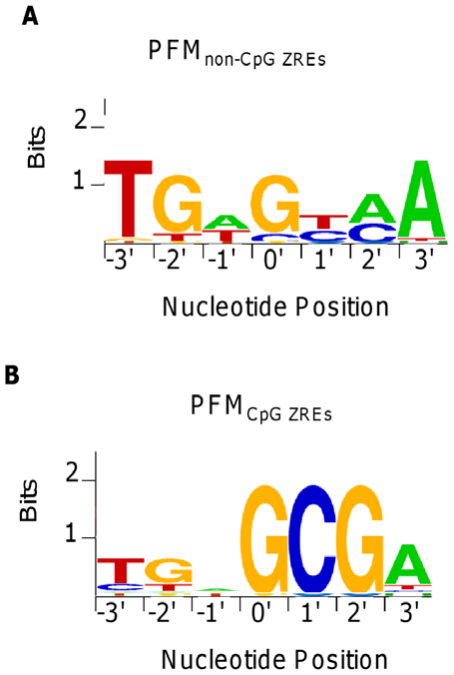
Sequence Logos CpG and non-CpG ZREs. The sequences comprising ZRE_32_, were divided into those sites which do not contain a CpG motif, class I ZREs and those that do, class II and class III ZREs. A. A PFM was created from (PFM_non-CpG ZREs_) and is displayed using relative letter height. B. A PFM was created from (PFM_CpG ZREs_) and is displayed using relative letter height.

The consensus binding sites identified for non-CpG ZREs are similar to the binding sites originally described for Zta (Figure 8). In contrast, the binding sites for CpG containing ZREs are remarkably different. This sequence is dominated by an almost invariant G 5′ to the absolute prerequisite for me-CpG at positions 1′ and 2′ in the right-half of the core sequence.

The identification of 58 EBV promoters that harbor methylation dependent CpG ZREs, combined with the knowledge that the EBV genome is heavily methylated during latency [3], [4], suggests that Zta plays an important role in overturning epigenetic silencing of over half of the EBV genes during lytic replication. Indeed, all three of the promoters tested displayed a strong interaction with Zta *in vivo* in ChIP analyses. A genome-wide DNA binding analysis was recently published identifying sequences to which a mutant form of Zta, that is replication and transactivation dead, can interact [3]. This report highlighted the strength of the interaction between Zta and methylation dependent binding of Zta to CpG ZREs in the EBV genome.

The EBV genes that contain only methylation-dependent ZREs are of particular interest. All of these genes are heavily methylated during viral latency yet unmethylated following replication and immediately after infection [3], [4]. Several are required for EBV replication and include components of the helicase/primase complex (*BBLF4*, *BBLF2/BBLF3*), the viral protein kinase (*BGLF4*), and glycoproteins gL (*BKRF2*) and gB (*BALF4*). In addition, the promoters for *BBLF4* and *BBLF2/BBLF3* have been validated as being targets for Zta that are completely dependent on methylation for Zta activation [3]. Our discovery that one in five EBV promoters contain CpG ZREs but have no methylation independent ZREs strongly supports the hypothesis that the unmethylated status of the EBV genome guards against the expression of the full range of lytic genes and therefore lytic replication during the establishment of latency.

Zta is the prototypic member of a family of transcription factors that interact with DNA in a methylation-dependent manner. C/EBP alpha has recently been shown to share the same characteristics [36]. It has been suggested that the interaction between C/EBP alpha and methylated sequence elements are needed to activate tissue specific genes during differentiation [36].

The biphasic methylation cycle is observed for several different classes of viruses that establish latency [4]. Yet even KSHV, which is closely related to EBV, does not contain a functional Zta homologue. The question arises as to how the methylated genomes of these viruses can be reactivated. We suggest that the recent discovery that a cellular transcription factor also has methylation dependent DNA binding properties [36] implies that other viruses may rely on host methylation dependent transcription factors to differentially control the expression of their genomes during the establishment of latency or replication.

## Methods

### Computational prediction of ZREs core sequences bound by Zta

The starting point for the computational approach was the Zta transcription factor entry T00923 in Transfac 8.3 that includes 6 experimentally verified ZRE binding sites [32], [33]. The Promo algorithm [37], [38] generated a position weight matrix (PWM) based on the T00923 transcription factor entry, and we used it to search 3 well-characterized Zta-responsive promoters from the EBV genome (*BZLF1* promoter (Zp) 500 bp upstream of the published transcription start sites were included. A positive match was taken as one with an 85% similarity rate.

### DNA binding assays

Electrophoretic mobility shift assays (EMSA) were undertaken using Zta protein generated in a wheat germ *in vitro* translation system (Promega) and [^32^P]-radio labeled double strand oligonucleotides, as described previously [39].

Where indicated in the figure, the central CpG motif was methylated on both cytosine residues during synthesis (Sigma) or methylated probes were synthesized or methylated *in vitro* using the CpG methyltransferase M.sssI (NEB) [23].

Zta protein (B95-8 strain) was *in vitro* translated using wheatgerm extract (Promega).

Zp (−96): TAAATTTAGGTGTGTCTATGAGGTACA
Zp (−365): ACAGATGGACCTGAGCCACCCGCC
Zp (−662): CCTCTTTGGCTGACACACCTCTCGCCC
Rp (−204) & RpZRE2: CATCTTGTCCTGTGATAAAATCGCTCATAAGCTTAGT
Rp (−439 & −447): ATGACTCGGGTGTGTCCTTGTGTGAGGTCTCACCTG
Rp (−114): CACTCATACTTAAGCGATGCTGATGCA
*BMRF1*p (−173): TGGGGGGTGGTGTGCCATACAAGGGAGC
*BMRF1*p (−248): CCTTGGTGGATGTGCGAGCCATAAAGCA
CpG5 A: AAGCGATGGCCGAGCGATGACTCGTGT
CpG5 B: TCCAGATGACTGAGCGCACGGCCTCAA
CpG5 C: AAAGATCTGGTTGGCGATCCGGTACAC
CpG5 D: TTGGAAAACATTAGCGACATTTACCTG
CpG5 E: TTTGGCGTCATGTGCGTCTGGATGACA
CpG5 F: CAGACTCTGGTTTGCGAGGCTGGGCGG
CpG5 G: GCCGCCGCACTCAGCGAGGAGGCCTGC
CpG5 H: CGAGGAGGCCTGCGCGTGTTCCTCAAC
CpG5 I: CCAATGTCTGCGTGCGAGCCGGGCTTG
CpG5 J: CTTTGCGCTCTGCGCGAGGACGAGCTC
CpG5 K: GCAGGGCCCCCGCGCGATCTAGGTAGG
CpG5 L: CTCATAGGTCAGAGCGACATAGAGGCG
CpG5 M: TTTCAAGTCGTGGGCGAATTAACTGAG
CpG5 N: AGCAAGGTGCTGGGCGTGGACCGCGCG
CpG5 O: CACTCATACTTAAGCGATGCTGATGCA
CpG5 P: GTCATGTAGGTGAGCGGGCAGTCCTTG

### Chromatin Immunoprecipitation

Chromatin was prepared from Akata cells [35], following induction with anti IgG, in the presence of 100 µM acyclovir essentially as described in [40], except that a mixture of Protein A and protein G were used to capture antibodies. Precipitation was undertaken using an amino-terminal Zta antibody from Santa Cruz.

Primers: absolute genomic position and sequence

OL5 F 41195-41216 CAGCTGACCGATGCTCGCCA
OL5 R 41342-41323 ATGGTGAGGCAGGCAAGGCG
BKRF4 F 98591-98611 CATTGCTCTCTGAGCGGTTA
BKRF4 R 98688-98667 ACCAGATGCTTCTTGGAGTTG
BGLF4 F 111611-111631 ACCGAGGCTCTTAGTTGCTG
BGLF4 R 111686-111666 GTTGCGGACATGGTGACTTA
BTRF1 F 126981-127000 AGCTACGCAATCGGAGTCA
BTRF1 R 127052-127034 GGAGGCGCAGTCTAGCAG

Region with no ZREs

OR1 F 139875-139895 CCGCATGTCCAACCACCACG
OR1 R 139997-139976 ATGCTACCTAGGCCTGCGTCC
OL1 F 38229-38249 GCGCAACAGTGCCACCAACC
OL1 R 38302-38282 CAGGACCTGGCGGTAGTGCAG
MID-BORF1 F 60768-60788 TGCCTGAGACCTCTCGGACGG
MID-BORF1 R 60817-60792 CCACGACGCAGTCCTTAGGATCATG

### Generation and application of ZRE PFMs

A position frequency matrix (PFM_CpG5_) was created using 5 CpG containing ZRE core binding sequences (Figure 3) and used to search (a) the 3 EBV promoters using the algorithm Matscan [41], and (b) the complete Human herpesvirus 4 (Epstein-Barr virus) Genome NC_007605 extracted from GenBank [42]. An 85% similarity score was used to define a positive match to the PFM_CpG5_. PFMs for non-CpG ZREs and CpG ZREs were generated in a similar manner and displayed using WEBLOGO [43].

Exact pattern matching was employed to search for each of the 32 core ZRE sequences within the EBV genome. A rolling window of seven nucleotides was used and an exact comparison of each of the core ZREs sequences, was made. In addition, the reverse complement of the sequence was checked in the same manner.

A MySQL database of the locations of the exact matches within the EBV genome was generated and simple analyses can be conducted using a web interface which is publically available at URL: http://bioinf.biochem.sussex.ac.uk/EBV/. The database uses gene annotations extracted from the RefSeq NC_007605 entry and data from the exact match predictions.

## Supporting Information

Table S1 The sequences of published ZREs used in this study are shown, together with their names and references. CpG motifs are shown in bold.
(DOCX)

Table S2 ZREs predicted using PROMO, together with the results of their evaluation by DNA binding assays (EMSA) are shown. CpG motifs are shown in bold.
(DOCX)

Table S3 All ZREs in the EBV genome are shown with the position of the central nucleotide using RefSeq NC_007605.
(DOCX)

Table S4 The number of ZREs close to each EBV promoter (between −1000 and +200 from the annotated gene start) are shown using RefSeq NC_007605.
(DOCX)
