# Supplementary material for: Epigenetic Control of Viral Life-Cycle by a DNA-Methylation Dependent Transcription Factor
Source: PLoS One. 2011 Oct 11;6(10):e25922. doi: 10.1371/journal.pone.0025922 (PMC3191170; doi:10.1371/journal.pone.0025922)
Supplement: Table S1 — The sequences of published ZREs used in this study are shown, together with their names and references. CpG motifs are shown in bold. (DOCX) [file pone.0025922.s001.docx]

**Table S1** Published ZREs used in this study. CpG motifs are highlighted in bold.

| **ZRE core sequence** | |  |  |  |
| --- | --- | --- | --- | --- |
| **Forward** | **Reverse** | **Class** | **Names** | **References** |
| TGAGCCA | TGGCTCA | I | Zp ZREIIIA | (4, 6, 10) |
| TGAGCCA | TGGCTCA | I | Rp ZRE1 | (9) |
| TTAGCAA | TTGCTAA | I | Zp ZREIIIB | (4, 6, 10) |
| TGTGTAA | TTACACA | I | DSL ZRE1 | (7) |
| TGAGCAA | TTGCTCA | I | DSL ZRE2 | (7) |
| TGAGCAA | TTGCTCA | I | DSL ZRE7 | (7) |
| TGAGCAA | TTGCTCA | I | BMRF1ZRE(-44) | (8) |
| TGAGCAA | TTGCTCA | I | BMRF1ZRE(-107) | (8) |
| TGTGTCA | TGACACA | I | DSL ZRE3 | (7) |
| TGTGTCA | TGACACA | I | DSL ZRE4 | (7) |
| TGTGTCA | TGACACA | I | DSL ZRE6 | (7) |
| TGTGCAA | TTGCACA | I | DSL ZRE5 | (7) |
| TGAGTCA | TGACTCA | I | BSLF2+BMLF1 | (3, 10) |
| TGAGTCA | TGACTCA | I | BMRF1 AP-1 | (8) |
| TGACTAA | TTAGTCA | I | Fp AP-1-Like Site | (5) |
| TGAG**CG**A | T**CG**CTCA | II | Rp ZRE2 | (1, 9) |
| TT**CGCG**A | T**CGCG**AA | III | Rp ZRE3 | (1) |
| CGGG**CG**A | T**CG**CCCG | III | Nap ZRE1 | (2) |
| TGAG**CG**T | A**CG**CTCA | III | Nap ZRE2 | (2) |

1. **Bhende, P. M., W. T. Seaman, H. J. Delecluse, and S. C. Kenney.** 2004. The EBV lytic switch protein, Z, preferentially binds to and activates the methylated viral genome. Nat Genet **36:**1099-104.

2. **Dickerson, S. J., Y. Xing, A. R. Robinson, W. T. Seaman, H. Gruffat, and S. C. Kenney.** 2009. Methylation-dependent binding of the epstein-barr virus BZLF1 protein to viral promoters. PLoS Pathog **5:**e1000356.

3. **Farrell, P., D. Rowe, C. M. Rooney, and T. Kouzarides.** 1989. Epstein-Barr virus BZLF1 trans-activator specifically binds to a consensus AP-1 site and is related to c-fos. EMBO J. **8:**127-132.

4. **Flemington, E., and S. H. Speck.** 1990. Autoregulation of Epstein-Barr Virus putative lytic switch gene BZLF1. J Virol **64:**1227-1232.

5. **Granato, M., A. Farina, R. Gonnella, R. Santarelli, L. Frati, A. Faggioni, and A. Angeloni.** 2006. Regulation of the expression of the Epstein-Barr virus early gene BFRF1. Virology **347:**109-16.

6. **Lieberman, P. M., and A. J. Berk.** 1990. In vitro transcriptional activation, dimerization, and DNA-binding specificity of the Epstein-Barr virus Zta protein. J. Virol. **64:**2560-8.

7. **Lieberman, P. M., J. M. Hardwick, J. Sample, G. S. Hayward, and S. D. Hayward.** 1990. The zta transactivator involved in induction of lytic cycle gene expression in Epstein-Barr virus-infected lymphocytes binds to both AP-1 and ZRE sites in target promoter and enhancer regions. J Virol **64:**1143-55.

8. **Quinlivan, E. B., E. A. Holley-Guthrie, M. Norris, D. Gutsch, S. L. Bachenheimer, and S. C. Kenney.** 1993. Direct BRLF1 binding is required for cooperative BZLF1/BRLF1 activation of the Epstein-Barr virus early promoter, BMRF1. Nucleic Acids Res **21:**1999-2007.

9. **Sinclair, A. J., M. Brimmell, F. Shanahan, and P. J. Farrell.** 1991. Pathways of activation of the Epstein-Barr virus productive cycle. J. Virol. **65:**2237-44.

10. **Urier, G., B. M., P. Chambard, and A. Sergeant.** 1989. The Epstein-Barr virus early protein EB1 activates trasncription from different responsive elements including AP-1 binding sites. EMBO J. **8:**1447-1453.
