# Supplementary material for: Epigenetic Control of Viral Life-Cycle by a DNA-Methylation Dependent Transcription Factor
Source: PLoS One. 2011 Oct 11;6(10):e25922. doi: 10.1371/journal.pone.0025922 (PMC3191170; doi:10.1371/journal.pone.0025922)
Supplement: Table S2 — ZREs predicted using PROMO, together with the results of their evaluation by DNA binding assays (EMSA) are shown. CpG motifs are shown in bold. (DOCX) [file pone.0025922.s002.docx]

**Table S2.** ZREs predicted using PROMO, together with the results of their evaluation by DNA binding assays (EMSA). CpG motifs in ZREs are shown in bold.

| **Promoter** | **Position** | **ZRE core sequence** | **EMSA Binding** | **Methylation-dependent** |
| --- | --- | --- | --- | --- |
| Zp | -96 | TGTGTCT | + | N/A |
| Rp | -204 | TGTGATA | - | N/A |
| Rp | -439 | TGTGTCC | - | N/A |
| Rp | -447 | TGTGTGA | - | N/A |
| BMRF1 | -173 | TGGCACA | + | N/A |
| BMRF1 | -248 | TGTG**CG**A | + | + |
