# Supplementary material for: Epigenetic Control of Viral Life-Cycle by a DNA-Methylation Dependent Transcription Factor
Source: PLoS One. 2011 Oct 11;6(10):e25922. doi: 10.1371/journal.pone.0025922 (PMC3191170; doi:10.1371/journal.pone.0025922)
Supplement: Table S3 — All ZREs in the EBV genome are shown with the position of the central nucleotide using RefSeq NC_007605. (DOCX) [file pone.0025922.s003.docx]

**Table S3** - All ZREs in EBV genome.

| **Sequence** | **Position** |
| --- | --- |
| TGAGCAA | 1064 |
| TGACACA | 1539 |
| TGACACA | 2496 |
| CCGCTCA | 2531 |
| ACGCTCA | 2809 |
| TGGCACA | 3659 |
| TGAGCAA | 3970 |
| TCGCTAA | 4274 |
| TCAGCGA | 4385 |
| TCGCCCA | 4595 |
| TCGCGAA | 4943 |
| TGTGCAA | 5851 |
| TGGCACA | 6496 |
| ACGCTCA | 6994 |
| TCAGCGA | 8055 |
| GCGCTCA | 8097 |
| TGTGTAA | 8114 |
| TGACTAA | 8916 |
| TTAGCAA | 9158 |
| TGTGTAA | 9299 |
| TGTGCAA | 9598 |
| GGAGCGA | 9676 |
| TGAGCAA | 9845 |
| TCGCCCA | 10488 |
| GGAGCGA | 11057 |
| TCGCAAA | 11101 |
| TCGCCCA | 11235 |
| TGGCACA | 12009 |
| GGAGCGA | 12119 |
| CGGGCGA | 12587 |
| CGGGCGA | 12613 |
| GGAGCGA | 13263 |
| CGGGCGA | 13334 |
| TCAGCGA | 14532 |
| TCGCCCA | 14733 |
| TGGCACA | 15081 |
| GGAGCGA | 15191 |
| CGGGCGA | 15659 |
| CGGGCGA | 15685 |
| GGAGCGA | 16335 |
| CGGGCGA | 16406 |
| TCAGCGA | 17604 |
| TCGCCCA | 17805 |
| TGGCACA | 18153 |
| GGAGCGA | 18263 |
| CGGGCGA | 18731 |
| CGGGCGA | 18757 |
| GGAGCGA | 19407 |
| CGGGCGA | 19478 |
| TCAGCGA | 20676 |
| TCGCCCA | 20877 |
| TGGCACA | 21225 |
| GGAGCGA | 21335 |
| CGGGCGA | 21803 |
| CGGGCGA | 21829 |
| GGAGCGA | 22479 |
| CGGGCGA | 22550 |
| TCAGCGA | 23748 |
| TCGCCCA | 23949 |
| TGGCACA | 24297 |
| GGAGCGA | 24407 |
| CGGGCGA | 24875 |
| CGGGCGA | 24901 |
| GGAGCGA | 25551 |
| CGGGCGA | 25622 |
| TCAGCGA | 26820 |
| TCGCCCA | 27021 |
| TGGCACA | 27369 |
| GGAGCGA | 27479 |
| CGGGCGA | 27947 |
| CGGGCGA | 27973 |
| GGAGCGA | 28623 |
| CGGGCGA | 28694 |
| TCAGCGA | 29892 |
| TCGCCCA | 30093 |
| TGGCACA | 30441 |
| GGAGCGA | 30551 |
| CGGGCGA | 31019 |
| CGGGCGA | 31045 |
| GGAGCGA | 31695 |
| CGGGCGA | 31766 |
| TCAGCGA | 32964 |
| TCGCCCA | 33165 |
| TGGCACA | 33513 |
| GGAGCGA | 33623 |
| CGGGCGA | 34091 |
| CGGGCGA | 34117 |
| GGAGCGA | 34767 |
| CGGGCGA | 34838 |
| TCGCCCA | 35355 |
| TGAGCCA | 35627 |
| TGGCACA | 36973 |
| TGAGTAA | 36999 |
| TGGCACA | 40268 |
| TGTGTAA | 40545 |
| TGAGCAA | 40563 |
| TGACACA | 40601 |
| TGACACA | 40634 |
| TGTGCAA | 41182 |
| TGAGCCA | 41299 |
| TGACACA | 41341 |
| TGAGCAA | 41446 |
| TGGCACA | 42064 |
| TGAGCCA | 42719 |
| TGACACA | 42745 |
| TGTGTAA | 42977 |
| TCGCTCA | 44488 |
| TGGCACA | 44931 |
| TGGCACA | 45013 |
| TGTGTAA | 45804 |
| TGGCACA | 46136 |
| TGAGCCA | 46229 |
| TGACACA | 46236 |
| TGACACA | 46649 |
| CGGGCGA | 46830 |
| GTTGCAA | 47938 |
| ACGCTCA | 48106 |
| TCGCGAA | 48276 |
| TGAGCCA | 48874 |
| CCGCTCA | 49180 |
| TGAGCCA | 49865 |
| TGACTAA | 49898 |
| TCGCAAA | 50112 |
| CGTGCGA | 50239 |
| TCAGCGA | 51867 |
| TCGCGCA | 52223 |
| CGTGCGA | 52313 |
| CCGCTCA | 53115 |
| TCGCTCA | 54329 |
| TCGCGCA | 54479 |
| CGGGCGA | 54867 |
| ACGCTCA | 55224 |
| GGAGCGA | 55612 |
| TCGCAAA | 55691 |
| TCGCGAA | 55922 |
| GGAGCGA | 55978 |
| CCGCTCA | 56000 |
| TGACACA | 56120 |
| AGAGCGA | 56585 |
| TCGCAAA | 56741 |
| AGAGCGA | 57203 |
| GGAGCGA | 57770 |
| TGACACA | 57916 |
| TCGCAAA | 58796 |
| TGAGTAA | 59231 |
| CGTGCGA | 59810 |
| CGGGCGA | 60129 |
| TGTGTAA | 60137 |
| CGGGCGA | 61602 |
| CCGCTCA | 62709 |
| TCGCGCA | 62739 |
| CGGGCGA | 62859 |
| TCAGCGA | 63099 |
| AGAGCGA | 63745 |
| TGAGTAA | 63836 |
| TGTGTAA | 64251 |
| TCGCGCA | 64356 |
| TCAGCGA | 64756 |
| TGGCACA | 64904 |
| GCGCTCA | 65095 |
| TGGCACA | 65510 |
| CGAGCGA | 65653 |
| ACGCACA | 66022 |
| CCGCTCA | 66379 |
| TGAGCAA | 66453 |
| TCGCACA | 67330 |
| TGGCACA | 67405 |
| CCGCTCA | 67430 |
| TGAGCAA | 67471 |
| TGACTCA | 67517 |
| TGAGCAA | 67534 |
| TGAGCCA | 68533 |
| AGACACA | 68865 |
| AGACACA | 68959 |
| TGACACA | 69642 |
| GCGCTCA | 69772 |
| TGTGTAA | 70425 |
| TGGCACA | 70771 |
| TTAGCAA | 70869 |
| TCGCTCA | 71579 |
| TGACTCA | 72140 |
| TGGCACA | 72260 |
| TGAGCAA | 72289 |
| TGGCACA | 72331 |
| TCGCTCA | 72357 |
| AGACACA | 72799 |
| TCGCTCA | 73151 |
| CGCGCGA | 73472 |
| ACGCACA | 73603 |
| GGAGCGA | 73674 |
| TAAGCGA | 73744 |
| ACGCACA | 73772 |
| TGTGCAA | 73895 |
| GCGCTCA | 74228 |
| TCGCTCA | 74259 |
| CGCGCGA | 74278 |
| CCGCTCA | 74313 |
| TGGCACA | 74474 |
| TTAGCAA | 74800 |
| GCGCTCA | 74961 |
| GCGCTCA | 75097 |
| TCGCTCA | 75398 |
| TGGCACA | 76293 |
| TGACTAA | 76489 |
| CCGCTCA | 77781 |
| TCGCAAA | 78712 |
| TCGCCAA | 78832 |
| TGAGCCA | 79610 |
| TGACACA | 79839 |
| TGACTCA | 79928 |
| TCGCCCA | 80217 |
| TCAGCGA | 80925 |
| AGAGCGA | 81114 |
| TGGCACA | 81283 |
| TCGCTAA | 81516 |
| CGGGCGA | 81523 |
| TGACACA | 81591 |
| TCGCTCA | 81676 |
| TGAGCCA | 82263 |
| TGACACA | 82824 |
| TGAGCCA | 83076 |
| TGACACA | 83158 |
| TGAGCAA | 83534 |
| TGACTCA | 83711 |
| AGACACA | 84759 |
| ACGCTCA | 85520 |
| TGACACA | 85672 |
| CGAGCGA | 85752 |
| TCGCCCA | 85961 |
| CCGCTCA | 86129 |
| TTAGCAA | 86512 |
| AGACACA | 86563 |
| TCGCCAA | 86656 |
| ACGCACA | 86722 |
| TTAGCAA | 87215 |
| GGAGCGA | 88057 |
| TGTGTAA | 88128 |
| TCGCCCA | 88189 |
| TCGCCCA | 88280 |
| CGTGCGA | 88298 |
| TGACTCA | 88879 |
| TGACACA | 89176 |
| ACGCACA | 90390 |
| AGACACA | 90998 |
| TTAGCAA | 91015 |
| TGAGCCA | 91028 |
| TGAGCCA | 91267 |
| TGACACA | 91564 |
| AGAGCGA | 91720 |
| AGAGCGA | 91867 |
| CCGCTCA | 92152 |
| ACGCTCA | 92675 |
| CGGGCGA | 92703 |
| ACGCACA | 93436 |
| TGAGCCA | 93923 |
| TAAGCGA | 94003 |
| TCGCTCA | 94083 |
| TCGCGAA | 94143 |
| CGTGCGA | 94635 |
| ACGCTCA | 95613 |
| AGACACA | 95714 |
| GGAGCGA | 96954 |
| GCGCTCA | 97772 |
| TCGCTAA | 97802 |
| TGAGCAA | 97820 |
| TCGCAAA | 98293 |
| CGTGCGA | 98544 |
| CCGCTCA | 98600 |
| TCGCTAA | 98776 |
| AGAGCGA | 99131 |
| TCGCGAA | 100070 |
| TCGCCCA | 100301 |
| ACGCTCA | 100875 |
| TCGCGAA | 101174 |
| AGAGCGA | 101557 |
| CGAGCGA | 102192 |
| TCGCACA | 102206 |
| ACGCTCA | 102328 |
| TCGCTCA | 102515 |
| GGAGCGA | 102573 |
| TGACTAA | 103356 |
| AGACACA | 104685 |
| AGACACA | 105007 |
| CGGGCGA | 105798 |
| TCGCAAA | 106516 |
| TCGCCCA | 106659 |
| TCGCTCA | 107508 |
| TCGCGCA | 107910 |
| TGAGTAA | 108242 |
| TGAGCCA | 109615 |
| TCGCCCA | 110120 |
| TGACTCA | 110187 |
| TCAGCGA | 110385 |
| CGAGCGA | 110422 |
| TGAGCAA | 110520 |
| GCGCTCA | 111586 |
| TAAGCGA | 111642 |
| ACGCTCA | 111830 |
| TGGCACA | 112019 |
| TGGCACA | 112041 |
| CGGGCGA | 112601 |
| TGAGCCA | 113412 |
| TGGCACA | 113561 |
| TTAGCAA | 113695 |
| TCGCTCA | 114508 |
| TGAGCAA | 115217 |
| TGGCACA | 115843 |
| TGAGTAA | 116169 |
| TCAGCGA | 116329 |
| AGACACA | 116442 |
| TGACACA | 117134 |
| TCAGCGA | 117301 |
| TGAGCCA | 117305 |
| ACGCACA | 117330 |
| TGACTCA | 117482 |
| TGACTAA | 117779 |
| TGAGCCA | 118010 |
| TGGCACA | 118770 |
| CCGCTCA | 118883 |
| TGACTAA | 119208 |
| AGAGCGA | 119628 |
| TGAGCCA | 119873 |
| CGGGCGA | 121488 |
| TGACTAA | 121678 |
| GCGCTCA | 121732 |
| TCGCCCA | 122177 |
| ACGCTCA | 122582 |
| CGTGCGA | 124443 |
| TGAGCAA | 124867 |
| TGACACA | 125177 |
| GCGCTCA | 125625 |
| TAAGCGA | 125682 |
| TGAGCCA | 125705 |
| GTTGCAA | 125960 |
| GTTGCAA | 125961 |
| AGAGCGA | 126320 |
| ACGCTCA | 126339 |
| TCGCCCA | 127124 |
| TGACACA | 127692 |
| CGGGCGA | 128065 |
| TCGCCAA | 128124 |
| TGGCACA | 128514 |
| TGGCACA | 128541 |
| CCGCTCA | 129105 |
| GGAGCGA | 129184 |
| TCAGCGA | 130682 |
| TGAGCAA | 130785 |
| TGACTCA | 131207 |
| CGGGCGA | 131551 |
| TGACTCA | 132419 |
| TAAGCGA | 132656 |
| TGAGCAA | 132865 |
| TGGCACA | 132939 |
| CGTGCGA | 132953 |
| TGAGCCA | 133577 |
| TCGCCAA | 134174 |
| TCGCCCA | 134722 |
| TGAGCCA | 134836 |
| CGTGCGA | 134890 |
| TGAGCCA | 135205 |
| CCGCTCA | 135309 |
| TCGCGCA | 135520 |
| CGCGCGA | 135588 |
| TGTGCAA | 135908 |
| TCGCCAA | 136720 |
| ACGCTCA | 136730 |
| TGAGCCA | 137354 |
| TGACACA | 137394 |
| TCGCAAA | 137420 |
| TGACTCA | 138228 |
| TCGCTAA | 138488 |
| TGAGCCA | 138537 |
| TGAGCAA | 138615 |
| TTAGCAA | 138682 |
| CGGGCGA | 139408 |
| AGACACA | 139577 |
| TGAGCCA | 140206 |
| TGTGCAA | 143463 |
| TGAGCAA | 143481 |
| TGTGTAA | 143553 |
| TGTGCAA | 144101 |
| TGAGCCA | 144218 |
| TGACACA | 144260 |
| TGTGCAA | 144875 |
| TGGCACA | 144985 |
| TGAGCAA | 145019 |
| TGTGCAA | 145048 |
| TGAGCCA | 145957 |
| TGGCACA | 146560 |
| ACGCACA | 147574 |
| GGAGCGA | 147692 |
| AGACACA | 147765 |
| TGGCACA | 148653 |
| CGCGCGA | 148804 |
| GCGCTCA | 149138 |
| AGAGCGA | 149824 |
| CGGGCGA | 150363 |
| CGAGCGA | 150440 |
| TCGCCCA | 150532 |
| CGTGCGA | 150576 |
| TGGCACA | 150639 |
| TGACTCA | 150945 |
| AGAGCGA | 151198 |
| TCGCCCA | 151207 |
| AGACACA | 151353 |
| AGACACA | 151359 |
| TGACACA | 151673 |
| TGAGCAA | 151806 |
| TGTGCAA | 151864 |
| GTTGCAA | 152777 |
| GGAGCGA | 152784 |
| GGAGCGA | 153004 |
| TCAGCGA | 153043 |
| TGTGTAA | 153679 |
| AGAGCGA | 153979 |
| TCGCTCA | 154723 |
| TCGCCCA | 155458 |
| TGACACA | 155644 |
| TGAGCAA | 156452 |
| TCGCTCA | 156537 |
| TGGCACA | 157098 |
| CCGCTCA | 158123 |
| TCGCGCA | 159076 |
| CGAGCGA | 159166 |
| ACGCACA | 159326 |
| AGAGCGA | 159547 |
| GCGCTCA | 159626 |
| CGGGCGA | 159847 |
| TGACTCA | 159919 |
| TCGCCCA | 160372 |
| ACGCTCA | 160846 |
| TCAGCGA | 161224 |
| TCGCCCA | 161241 |
| TGTGCAA | 161262 |
| TGGCACA | 161974 |
| CCGCTCA | 162449 |
| AGAGCGA | 162946 |
| TCGCCCA | 163069 |
| CGTGCGA | 163432 |
| GGAGCGA | 163832 |
| TGAGTAA | 164363 |
| TCAGCGA | 164458 |
| TCGCAAA | 164485 |
| TCGCCCA | 164520 |
| TCGCTCA | 165058 |
| TCGCTCA | 165122 |
| TCGCCCA | 165367 |
| ACGCTCA | 165400 |
| TGAGCCA | 165516 |
| GTTGCAA | 166363 |
| TGACTCA | 166416 |
| TCGCTCA | 167108 |
| TGACACA | 167192 |
| TGGCACA | 167265 |
| TGGCACA | 167602 |
| TGACTAA | 167701 |
| AGAGCGA | 168648 |
| TGAGTAA | 168744 |
| TGACACA | 169027 |
| TCGCACA | 169590 |
| AGACACA | 169878 |
| TGACACA | 170114 |
| AGACACA | 170416 |
| TGACACA | 170652 |
| AGACACA | 170939 |
| TGACACA | 171175 |
| AGACACA | 171477 |
| TGACACA | 171713 |
