# Supplementary material for: Epigenetic Control of Viral Life-Cycle by a DNA-Methylation Dependent Transcription Factor
Source: PLoS One. 2011 Oct 11;6(10):e25922. doi: 10.1371/journal.pone.0025922 (PMC3191170; doi:10.1371/journal.pone.0025922)
Supplement: Table S4 — The number of ZREs close to each EBV promoter (between −1000 and +200 from the annotated gene start) are shown using RefSeq NC_007605. (DOCX) [file pone.0025922.s004.docx]

**Table S4.** Number of ZREs within EBV promoters.

| **Promoter**  **name** | **Class I** | **Class II** | **Class III** | **Total** |
| --- | --- | --- | --- | --- |
| BNRF1 | 1 | 0 | 0 | 1 |
| EBER 1 | 1 | 0 | 0 | 1 |
| EBER 2 | 1 | 0 | 1 | 2 |
| BCRF1 | 3 | 0 | 1 | 4 |
| Cp | 0 | 0 | 3 | 3 |
| BWRF1 | 0 | 0 | 3 | 3 |
| Wp | 0 | 0 | 1 | 1 |
| BHLF1 | 4 | 0 | 0 | 4 |
| BHRF1 | 4 | 0 | 0 | 4 |
| BFLF2 | 2 | 0 | 0 | 2 |
| BFRF1A | 4 | 0 | 0 | 4 |
| BFLF1 | 4 | 0 | 0 | 4 |
| BFRF1 | 4 | 0 | 0 | 4 |
| BFRF2 | 0 | 0 | 0 | 0 |
| BFRF3 | 1 | 0 | 1 | 2 |
| Qp | 2 | 0 | 2 | 4 |
| BPLF1 | 1 | 0 | 2 | 3 |
| BORF1 | 0 | 0 | 3 | 3 |
| BOLF1 | 0 | 0 | 2 | 2 |
| BORF2 | 1 | 0 | 1 | 2 |
| BaRF1 | 1 | 0 | 2 | 3 |
| BMRF1 | 4 | 0 | 2 | 6 |
| BMRF2 | 1 | 0 | 0 | 1 |
| BSLF2/BMLF1 | 4 | 1 | 0 | 5 |
| BSLF1 | 2 | 1 | 0 | 3 |
| BSRF1 | 1 | 2 | 2 | 5 |
| BLRF1 | 1 | 0 | 0 | 1 |
| BLLF3 | 2 | 0 | 0 | 2 |
| BLRF2 | 2 | 0 | 0 | 2 |
| BLLF2 | 0 | 0 | 1 | 1 |
| BLLF1 | 2 | 0 | 1 | 3 |
| BZLF2 | 0 | 0 | 0 | 0 |
| BZLF1 | 4 | 0 | 0 | 4 |
| BRRF1 | 0 | 0 | 2 | 2 |
| BRLF1 | 1 | 1 | 2 | 4 |
| BRRF2 | 1 | 1 | 2 | 4 |
| BKRF2 | 0 | 1 | 1 | 2 |
| BKRF3 | 1 | 1 | 1 | 3 |
| BKRF4 | 0 | 0 | 3 | 3 |
| BBRF1 | 0 | 0 | 2 | 2 |
| BBLF4 | 0 | 0 | 3 | 3 |
| BBRF2 | 1 | 0 | 0 | 1 |
| BBRF3 | 0 | 0 | 2 | 2 |
| BBLF2/BBLF3 | 0 | 0 | 1 | 1 |
| BBLF1 | 0 | 0 | 0 | 0 |
| BGLF5 | 2 | 0 | 3 | 5 |
| BGLF4 | 0 | 1 | 1 | 2 |
| BGLF3.5 | 2 | 0 | 2 | 4 |
| BGRF1/BDRF1 | 0 | 0 | 1 | 1 |
| BGLF3 | 0 | 0 | 0 | 0 |
| BGLF2 | 0 | 1 | 0 | 1 |
| BGLF1 | 2 | 0 | 1 | 3 |
| BDLF4 | 1 | 0 | 0 | 1 |
| BDLF3.5 | 3 | 0 | 2 | 5 |
| BDLF3 | 2 | 0 | 1 | 3 |
| BDLF2 | 0 | 0 | 0 | 0 |
| BDLF1 | 0 | 0 | 1 | 1 |
| BcRF1.2 | 2 | 0 | 0 | 2 |
| BcLF1 | 1 | 1 | 1 | 3 |
| BTRF1 | 0 | 0 | 1 | 1 |
| BXLF2 | 1 | 0 | 0 | 1 |
| BXRF1 | 1 | 0 | 1 | 2 |
| BXLF1 | 2 | 0 | 2 | 4 |
| BVRF1 | 2 | 0 | 2 | 4 |
| BVRF2 | 1 | 0 | 3 | 4 |
| BVLF1 | 1 | 0 | 2 | 3 |
| BdRF1 | 1 | 0 | 0 | 1 |
| BILF2 | 4 | 0 | 1 | 5 |
| RPMS1 | 2 | 0 | 1 | 3 |
| LF3 | 2 | 0 | 0 | 2 |
| LF2 | 1 | 0 | 4 | 5 |
| LF1 | 3 | 0 | 0 | 3 |
| BILF1 | 1 | 0 | 3 | 4 |
| A73 | 1 | 0 | 1 | 2 |
| BALF5 | 1 | 1 | 0 | 2 |
| BALF4 | 0 | 0 | 3 | 3 |
| BARF0 | 0 | 0 | 2 | 2 |
| BALF3 | 1 | 0 | 3 | 4 |
| BALF2 | 1 | 0 | 3 | 4 |
| BALF1 | 0 | 2 | 2 | 4 |
| BARF1 | 0 | 2 | 1 | 3 |
| LMP-2A | 1 | 0 | 0 | 1 |
| BNLF2b | 2 | 1 | 0 | 3 |
| BNLF2a | 2 | 1 | 0 | 3 |
| LMP-1 | 1 | 0 | 0 | 1 |
| LMP-2B | 1 | 0 | 0 | 1 |
